# Methanol extract from Vietnamese *Caesalpinia sappan* induces apoptosis in HeLa cells

**DOI:** 10.1186/0717-6287-47-20

**Published:** 2014-05-27

**Authors:** Tran Manh Hung, Nguyen Hai Dang, Nguyen Tien Dat

**Affiliations:** Faculty of Chemistry, University of Science, Vietnam National University-HoChiMinh City, 227 Nguyen Van Cu, District 5, HoChiMinh City, Vietnam; Institute of Marine Biochemistry, Vietnam Academy of Science and Technology (VAST), 18 Hoang Quoc Viet, Caugiay District, Hanoi, Vietnam

**Keywords:** *Caesalpinia sappan*, Leguminosae, MTT assay, DNA fragmentation, Caspase-3

## Abstract

**Background:**

This study evaluated the cytotoxic activity of extracts from *Caesalpinia sappan* heartwood against multiple cancer cell lines using an MTT cell viability assay. The cell death though induction of apoptosis was as indicated by DNA fragmentation and caspase-3 enzyme activation.

**Results:**

A methanol extract from *C. sappan* (MECS) showed cytotoxic activity against several of the cancer cell lines. The most potent activity exhibited by the MECS was against HeLa cells with an IC_50_ value of 26.5 ± 3.2 μg/mL. Treatment of HeLa cells with various MECS concentrations resulted in growth inhibition and induction of apoptosis, as indicated by DNA fragmentation and caspase-3 enzyme activation.

**Conclusion:**

This study is the first report of the anticancer properties of the heartwood of *C. sappan* native to Vietnam. Our findings demonstrate that *C. sappan* heartwood may have beneficial applications in the field of anticancer drug discovery.

## Background

Apoptosis is a form of cell death that occurs in organisms under pathological conditions and contributes to cell replacement, tissue remodeling, and removal of damaged cells under normal conditions [1]. Therapies designed to enhance or decrease the susceptibility of individual cell types to apoptosis could provide a basis for the treatment of a variety of human diseases [2]. However, inappropriate regulation of apoptosis can cause serious disorders, such as neural degeneration, autoimmune disease, and cancer [3, 4]. Natural products with apoptotic activity have attracted a great deal of attention as potential new sources of leads for anti-cancer drugs. Various phytochemicals from medicinal plants have been shown to exert antitumoric effects via apoptosis.

*Caesalpinia sappan* L. (Leguminosae) is widely distributed in Southeast Asia. It has been used as an herbal medicine for treatment of inflammation and improvement of blood circulation [5], and as an anti-influenza, anti-allergic, and neuroprotective medication [6–8]. *C. sappan*, called “Tô Mộc” in Vietnam, is dispersed across low mountainous areas, requires high light, and is drought-tolerant. “Tô Mộc” was used in Vietnamese traditional medicine for treatment of menstrual and postpartum hematometra, trauma blood static, dizziness, and postpartum blood losses. It was also used in therapy for bloody dysentery, enteralgia, intestinal hemorrhage, and infectious diarrhea and for washing wounds [9]. The major active components in *C. sappan* are phenolics of four structural subtypes: brazilin, chalcone, protosappanin, and homoisoflavonoids [10–15]. An ethanol extract of *C. sappan* ameliorated hypercholesterolemia in C57BL/6 mice and suppressed inflammatory responses in human umbilical vein endothelial cells (HUVECs) through an antioxidant mechanism [11]. Compounds with a sappanchalcone skeleton exhibited antiinflammatory, antibacterial, and antiinfluenza activities [12–15]. Nguyen et al. reported that a methanol extract and the active compounds from *C. sappan* heartwood collected in Vietnam exhibited significant xanthine oxidase (XO) inhibitory activity [16, 17]. However, limited information is available concerning the anticancer activity of *C. sappan* originated from Vietnam*.* In this study, we investigated *in vitro* the anticancer properties, including antiproliferation activity and apoptosis induction, of a methanol extract from Vietnamese *C. sappan* heartwood.

## Results and discussion

The methanol, ethanol, and water extract yields from *C. sappan* heartwood were 18.8, 20.5, and 15.6%, respectively (Table 1). Multiple cancer cell lines were used to evaluate the potential inhibitory effects of these extracts on cell growth. Cancer cells were seeded in 96-well plates, incubated for 4 h, and then treated with various concentrations (5–100 μg/mL) of the extracts or with the standard anticancer drug, adryamicin. Cytotoxic effects were assessed using an MTT cell viability assay [18]. The methanol and ethanol extracts showed cytotoxic activity against HeLa cells with IC_50_ values of 26.5 ± 3.2 and 39.2 ± 3.0 μg/mL, respectively (Table 2). The methanol extract also exhibited significant cytotoxic activity against HL-60, MCF-7, HepG2, and KB cancer cells with IC_50_ values of 40.7 ± 2.8, 37.7 ± 1.1, 65.1 ± 3.5, and 76.7 ± 4.1 μg/mL, respectively. The ethanol extract displayed cytotoxicity against HL-60 and LLC cells with IC_50_ values of 68.5 ± 5.1 and 39.2 ± 2.0 μg/mL, respectively. The water extract showed weak cytotoxicity against all cancer cell lines, except for HeLa cells, with an IC_50_ value of 37.8 ± 3.6 μg/mL (Table 2). Because the methanol extract (MECS) exhibited the most potent cytotoxic activity, it was selected for further evaluation of its inhibition of HeLa cell proliferation. Cells were treated with MECS (5–100 μg/mL), incubated for 7 days, and counted at 2-day intervals using the trypan blue exclusion method. The growth of cells treated with MECS (10 μg/mL) was significantly inhibited by 38.4 ± 5.2% after 3 days relative to control cells. When the concentration of MECS was increased to 20, 50, or 100 μg/mL, the percentage of inhibition was increased to 78.7 ± 6.5, 86.4 ± 3.6, or 95.8 ± 1.0%, respectively, after 7 days of incubation (data not shown).

**Table 1:** **Percentage yields of extracts (% w/w)**

Plant extracts	Percentage yield (%)^a^
MeOH ex.	18.8 ± 5.4
EtOH ex.	20.5 ± 4.7
Water ex.	15.6 ± 7.5

^a^Data are presented as the mean ± SD of results from three independent experiments.

**Table 2:** **Cytotoxic activity of extracts from** ***C. sappan***

Extracts	IC_50_value (μg/mL)^a^							
	HL-60	HeLa	MCF7	LLC	HepG2	KPL4	HT-29	KB
MeOH ex.	40.7 ± 5.8	26.5 ± 3.2	37.7 ± 3.1	> 100	65.1 ± 3.5	> 100	> 100	76.7 ± 4.8
EtOH ex.	68.5 ± 5.1	39.2 ± 2.0	> 100	25.1 ± 3.8	> 30	> 100	> 100	> 100
Water ex.	> 100	37.8 ± 3.6	> 100	> 100	78.6 ± 4.3	> 100	> 100	> 100
Camptothecin^b^	6.2 ± 0.2	3.4 ± 0.2	4.5 ± 0.3	5.1 ± 0.7	6.2 ± 0.4	14.5 ± 1.7	10.7 ± 0.8	4.5 ± 0.4

^a^The inhibitory effects are represented as giving 50% inhibition (IC_50_) relative to the vehicle control. These data represent the average values of three repeated experiments (mean ± S.D.).

^b^Positive control.

**Figure 1:**
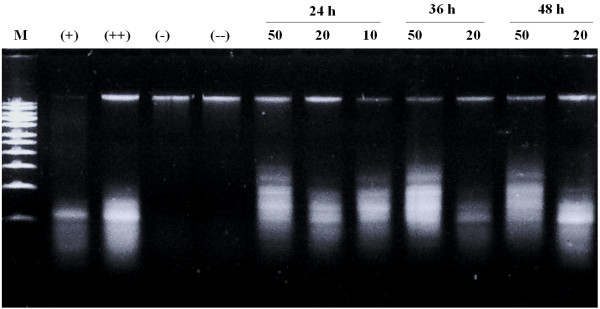
**Induction of the DNA fragmentation in HeLa cells** ***in vitro*** . HeLa cells were treated with MECS for 24 h (50, 20, 10 μg/mL), and for 36 and 48 h (50, 20 μg/mL). Total genomic DNA was extracted and resolved on a 1% agarose gel. Apoptotic DNA fragmentation was visualized by ethidium bromide staining. M, size marker; (−), mature cell; (−−), 0.1% DMSO-treated cells; (+), positive control treated with 2.5 μg/mL and (++), 5 μg/mL camptothecin.

To evaluate whether the inhibition of HeLa cell growth by MECS was mediated by apoptosis, we performed a DNA laddering assay [19]. Chromosomal DNA extracted from cells treated with 10, 20, or 50 μg/mL MECS for 24 h exhibited internucleosomal DNA fragmentation consisting of multiple fragments approximately 180–200 base pairs in size as visualized by agarose gel electrophoresis. At higher MECS concentrations, 20 and 50 μg/mL, internucleosomal DNA fragmentation was also observed after 36- and 48-h incubations (Figure 1).

**Figure 2:**
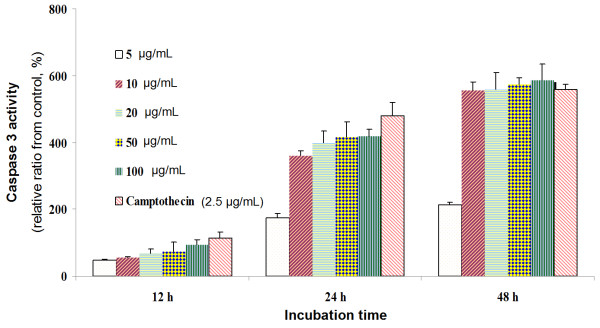
**The increment of caspase-3 activity in HeLa cells** ***in vitro.*** After 12, 24 and 48 h incubation with MECS, HeLa cells lysates were incubated at 37°C with caspase-3 substrate (Ac-DEVD-AFC) for 1 h. The fluorescence intensity of the cell lysates was measured to determine the caspase-3 activity. The blank group was used as 0.1% DMSO-treated cells; Camptothecin (2.5 μg/mL) was used as positive control. Data are presented as the mean ± S.D. of results from three independent experiments (* *P* < 0.05 vs. control).

Caspase-3 is a cytosolic protein that normally exists as a 32-kDa inactive precursor, which undergoes proteolytic cleavage into a heterodimer during apoptosis. Caspase-3 activity was determined by the amount of proteolytic cleavage of Ac-Asp-Glu-Val-Asp-8-amino-4-trifluoromethylcoumarin (Av-DEVD-AFC) [18] in HeLa cells after 12, 24, or 48 h treatment with MECS (5, 10, 20, 50, or 100 μg/mL). The caspase-3 activity increased by one- to six-fold in dose- and time-dependent manner relatives to the control (Figure 2).

Induction of apoptosis is a useful strategy in cancer therapies and is an important property of candidate anticancer drugs that distinguishes them from toxic compounds. Much effort has been directed into the search for compounds that influence apoptosis and to understanding their mechanisms of action. In this study, we showed that MECS exhibited a potent inhibitory activity against various cancer cell lines, especially against HeLa cells, through the induction of apoptosis. The *C. sappan* extract caused cell death by increasing the duration of the sub-G1 phase of the cell cycle and the condensation and shrinkage of nuclei in HNSCC4 and HNSCC31 (head and neck) cancer cell lines [20]. Among the compounds isolated from *C. sappan*, isoliquiritigenin 2’-methyl ether inhibited the growth of oral cancer cells via a pathway involving MAP kinases, NF-κB, and Nrf2 [21]. Sappanchalcone, a flavonoid, suppressed oral cancer cell growth and induced apoptosis through the activation of p53-dependent mitochondrial p38, ERK, JNK, and NF-κB signaling [22]. Brazilein, a major phenolic compound from *C. sappan*, exhibited antioxidant activity, inhibited intracellular lipid accumulation during adipocyte differentiation in 3 T3-L1 cells, and suppressed the induction of peroxisome proliferator-activated receptor γ [23]. The toxic effects of brazilein were evaluated in terms of cell viability, induction of apoptosis, and caspase-3 activity in BCC cells [23]. Brazilein also showed dose-dependent inhibition of cell proliferation and induction of apoptosis in glioma cells by increasing the proportion of cleaved poly-(ADP)-ribose polymerase and by decreasing caspase-3 and caspase-7 expression [24].

This study showed that addition of methanol (MECS), ethanol, and water extracts of *C. sappan* heartwood to growth medium decreased the HeLa cell population. Treatment with MECS induced apoptosis, thereby inhibiting HeLa cell growth. Further biochemical analysis indicated that the inhibitory activity of MECS against proliferation was related to the induction of apoptosis. Cultured HeLa cells treated with MECS (5–100 μg/mL) exhibited morphological features typical of apoptosis, which was consistent with the dose- and time-dependent DNA fragmentation, as determined by agarose gel electrophoresis (Figure 1). Several caspases play important roles in the regulation of apoptosis. They are grouped broadly as initiator or effector caspases according to the roles they play in inducing the apoptotic system. The initiator caspases include caspase-1, −8, and −9. Caspase-8 and −9 are typically activated by two alternative pathways. The first involves cell death receptor-mediated apoptosis via caspase-8 and is characterized by binding of cell death ligands and cell death receptors and subsequent activation of caspase-8 and −3. The second pathway involves mitochondria-mediated apoptosis via caspase-9. In both pathways, caspase-3 activation plays a central role in the initiation of apoptosis [25]. MECS concentrations of 5–100 μg/mL induced caspase-3 activation in dose- and time-dependent manners (Figure 2). Many models of apoptosis include loss of mitochondrial transmembrane potential mediated by opening of the mega-channel, which precedes caspase activation. Caspase-3 activation is required for several typical hallmarks of apoptosis and is indispensable for apoptotic chromatin condensation and DNA fragmentation.

## Conclusion

This study for the first time determined that MECS effectively inhibit the proliferation of HeLa cells by mechanism involved the induction of apoptosis. According to these results, it is suggested that the methanol extract of Vietnamese *C. sappan* may be a considerable source for the development of anti-cancer drugs. Further investigation to determine its bioactive components is currently in progress.

## Methods

### Plant material

The heartwood of *Caesalpinia sappan* L. was collected in An Giang province, Vietnam, in September 2010. Professor Tran Cong Luan at Vietnam National Institute of Medicinal Material botanically authenticated the plant. A voucher specimen number TCL-00120 describing the plant was deposited at the institute.

### Extract preparation

The extracts were prepared according to World Health Organization protocol (CG-1983) with a slight modification. The plant was dried and powdered, 50 g powder was extracted with 500 mL of methanol, ethanol or distilled water (3 times) for 3 hours under reflux. After extractions, the extracts were combined and filtered with Whatman filter paper No.1, and then were concentrated *in vacuo* to dryness. All extracts were stored at −4°C until use.

### Cell lines and culture

HL-60 (human promyelocytic leukemia), HeLa (human cervical adenocarcinoma), MCF-7 (human breast adenocarcinoma), LLC (lewis lung carcinoma), HepG2 (liver hepatocellular cells), KPl4 (human breast cancer), HT-29 (human colon carcinoma) and KB (mouth epidermal carcinoma) cells were obtained from the American Type Culture Collection (ATCC). The cells were maintained in RPMI or IMDM (GibcoBRL, NY, USA) with 10% fetal bovine serum (FBS) supplemented with 2% penicillin and 100 μg/mL of streptomycin at 37°C in a humidified atmosphere containing 5% CO_2_.

### Cytotoxic activity assay

The cancer cell lines were maintained in RPMI 1640 or IMDM that included L-glutamine (GIBCO) with 10% FBS (GIBCO) and 2% penicillin-streptomycin (GIBCO). Cells were cultured at 37°C in a 5% CO_2_ incubator. Cytotoxic activity was measured using a modified MTT assay [18]. Viable cells were seeded in the growth medium into 96-well microtiter plates (1 × 10^4^ cells/well) and were incubated at 37°C in a 5% CO_2_ incubator. A test sample was dissolved in DMSO and was adjusted to final sample concentrations ranging from 5 to 100 μg/mL by diluting with the growth medium. Each sample was prepared in triplicate. The final DMSO concentration was adjusted to <0.1%. After standing for 24 h, the test sample was added to each well. The same volume of medium with 0.1% DMSO was added to the control wells. 48 h after the addition of the test sample, MTT reagent was added to each well (final concentration: 5 μg/mL). 4 h later, the plate was centrifuged for 5 min at 1500 rpm, the medium was removed, and the resulting formazan crystals were dissolved in DMSO. The optical density (OD) was measured at 570 nm using a Titertek microplate reader (Multiskan MCC/340, Flow). The IC_50_ value was defined as the concentration of sample which reduced absorbance by 50% relative to the vehicle-treated control.

### DNA gel electrophoresis (DNA laddering)

DNA from the cells was purified by using an Apoptotic DNA Ladder Kit (Roche, Germany), and DNA bands were photographed under ultraviolet illumination [19]. HeLa cells (5 × 10^5^ cell/mL) were treated with the indicated concentration of tested extract for 24 h. After the supernatant was removed by centrifugation (1500 rpm, 4°C), the cells were washed with 1 mL of PBS and was precipitated by centrifugation at 3000 rpm for 10 min at 4°C. DNA from the cells was purified by using an Apoptotic DNA Ladder Kit and was separated on 1% (w/v) agarose gel containing 2.5 μL of 10 mg/mL ethidium bromide in TBE buffer [0.045 M Tris-borate (pH 8.0), 0.001 M EDTA].

### Caspase-3 activity

Caspase-3 enzyme activity was measured by proteolytic cleavage of the fluorogenic substrate Ac-DEVD-AFC by counting on a fluorescence plate reader (Twinkle LB970 microplate fluorometer, Berthold Technologies, Germany). HeLa cells (1 × 10^5^ cell/well) were treated with plant extract at concentrations of 5, 12.5, 25, 50 and 100 μg/mL. After incubation for 24 h, cells were harvested and washed with cold PBS. The pellets were lyzed using 15 μL of lysis buffer [10 mM Tris–HCl (pH 8.0), 10 mM EDTA, 0.5% Triton X-100] at room temperature for 10 min, and then placed on ice; 100 μL of assay buffer [100 mM Hepes (pH 7.5), 10 mM dithiothreitol, 10% (w/v) sucrose, 0.1% (v/v) Chaps, 0.1% (v/v) BSA] and 10 μL of substrate solution (200 μM substrate in assay buffer) were added. After incubation at 37°C for 1 h, fluorescence was measured with excitation at 370 nm and emission at 505 nm [18].

### Statistical analysis

The results are expressed as mean value ± S.D. Statistical analysis was performed using one-way ANOVA. A *P* < 0.05 was considered statistically significant.
